# Screening travellers to high-endemic countries for infection with *Mycobacterium tuberculosis* using interferon gamma release assay; a prospective study

**DOI:** 10.1186/1471-2334-14-515

**Published:** 2014-09-23

**Authors:** Floor Elfrink, Anneke van den Hoek, Marlies E Mensen, Gerard JB Sonder

**Affiliations:** Department of Infectious Diseases, Public Health Service (GGD), Nieuwe Achtergracht 100, 1018 WT Amsterdam, The Netherlands; National Coordination Centre for Traveller’s Health Advice (LCR), Amsterdam, The Netherlands; Department of Internal Medicine, Division of Infectious Diseases, Tropical Medicine and AIDS, Amsterdam, The Netherlands

## Abstract

**Background:**

International travel from low-incidence to high-incidence countries for tuberculosis (TB) is regarded as a risk factor for acquiring TB infection. In this prospective study among long-term travellers we examined the incidence of TB infection using Interferon gamma release assay (IGRA) test and compared these data with results from a visit to the TB department to which all long-term travellers were routinely referred.

**Methods:**

Immunocompetent adults, travelling for 13–52 weeks to TB-endemic countries, donated blood pre- and post-travel for IGRA. The pre-travel IGRA was only tested in case of a positive IGRA post-travel. Results from their visit(s) to the TB department for TST pre- and post-travel were collected and compared with study results.

**Results:**

We found two IGRA conversions in a group of 516 travellers, resulting in an attack rate (AR) of 0.4% (95% CI: 0.5 - 13.9) and an incidence rate (IR) of 0.85 per 1000 person-months (95% CI: 0.1-3.1).

We found 5 tuberculin skin test (TST) conversions, resulting in AR of 1.9% (5/261; 95% CI: 0.6 - 4.4) and an IR of 4.26 per 1000 person-months (95% CI: 1.38- 9.94). In our study these converters all had a negative IGRA. One traveller however, who was retested later at the TB department due to a positive TST, then appeared to have seroconverted.

**Conclusions:**

The risk of long-term travellers among our study population acquiring TB infection is low. We conclude that post-travel IGRA alone could be used for screening for TB infection among long-term travellers to high-endemic TB countries, but preferably not earlier than 8 weeks after return. One might even argue that IGRA testing should be limited to only those travellers who are going to work in a medical setting. A person with a positive IGRA should be referred to a TB physician for further evaluation.

**Electronic supplementary material:**

The online version of this article (doi:10.1186/1471-2334-14-515) contains supplementary material, which is available to authorized users.

## Background

International travel from low-incidence to high-incidence countries for tuberculosis (TB) is regarded as a risk factor for acquiring TB infection. This risk increases with the time spent in a high-incidence country and travellers’ participation in high-risk activities such as working in healthcare settings [1, 2].

A latent TB infection (LTBI) can progress to active, infectious disease. For the majority, the estimated lifetime risk of developing active TB for individuals with an LTBI is 10% within the first years after infection [3, 4]. Identifying and treating people with (recently acquired) LTBI is important in disease control, especially in countries with a low incidence of TB.

For a long time the only available test for detecting *Mycobacterium tuberculosis* infection was the tuberculin skin test (TST). TST has several limitations: intra-observer variation in reading the results, limited specificity in BCG-vaccinated individuals, false-positive results due to non-tuberculous mycobacteria, and a boosting phenomenon if TST is repeated. In addition, TST must be read after three days, requiring a second healthcare visit, thus resulting in poor compliance [5].

Interferon gamma release assay (IGRA), commercially available since 2005, is an in vitro T-cell based test which quantifies the immune response of T-cells to in vitro exposure to antigens of *M. tuberculosis*, other than those used in BCG vaccination [6]. A positive test result suggests *M. tuberculosis* infection. Compared to TST, IGRA has several advantages: only a single patient visit is required, results can be available within 24 hours, and there is no cross reaction with the BCG vaccine or with most non-tuberculous mycobacteria.

A gold standard for diagnosis of TB infection is absent, hindering good evaluation of the tests. A review in 2008 investigating the role of IGRAs in screening of travellers, showed too many limitations to come to a recommendation. Thus, the advantage of screening travellers by use of IGRA remains unclear [4].

A previous Dutch study conducted in 1994–1996 demonstrated that the incidence of TB for long-term travellers (3–12 months) based on TST conversions approaches that of the local population when travelling to high-incidence countries [1]. In this prospective study among long-term travellers we examined the incidence of TB infection using IGRA and we compared IGRA results with data from the TB department to which long-term travellers were referred according to national traveller guidelines.

## Methods

### Study population

This study was part of a larger prospective mono-centre study among persons attending the Public Health Service (PHS) Amsterdam travel clinic in the Department of Infectious Diseases [7]. All immunocompetent persons ≥18 years and of Dutch or other Western ethnicity were eligible if they were planning to travel to (sub)tropical countries for 13–52 weeks. For this sub-study, only participants who spent ≥13 weeks in a TB-endemic country, defined as countries with an annual TB incidence of >50/100,000, were included [8].

Before departure, a standard questionnaire was used to collect data on socio-demographics, BCG-vaccination history, travel history (indicated as cumulative time spent in Asia, Latin America, or Africa during lifetime), and travel purpose. The study protocol was approved by the Medical Ethics Committee of the Academic Medical Center Amsterdam. Participants were included after providing written informed consent.

Blood samples were taken before and 2–6 weeks after travel.

For IGRA the QuantiFERON-TB Gold (Cellestis Limited, Victoria, Australia) was performed following the manufacturer’s instruction and was considered positive if ≥ cut-off point of 0.35 IU/ml. All post-travel samples were tested. Due to financial constraints only in case of a positive post-travel sample the pre-travel sample was tested, which had been stored at −80°C. Positive post-travel samples with negative pre-travel samples were considered conversions.

TST was performed according to the Mantoux method with 0.1 ml Purified Protein Derivative (PPD) RT23 (Statens Serum Institute, Copenhagen, Denmark). Experienced medical staff of the TB department read the induration between 72 hours and 96 hours. Differences in induration of ≥10 mm between pre- and post-travel TST were considered conversions if pre-travel TST was <10 mm.

Although TST is recommended ≥ 8 weeks post-travel, participants in this study were referred to the TB department for a TST at the same time as the post-travel study visit. At the TB department, they were either tested and/or asked to return 8 weeks after travel.

Data analysis was performed with SPSS version 19.0.0.1 (2010, IBM, Somers, USA).

Chi-square tests were used to evaluate predictive value for TST or IGRA conversion. Tested characteristics included: age, sex, BCG, reason for travel, healthcare work, travel duration, and primary destination.

Incidences were expressed as the rate based on the person-time spent in endemic countries. Person-time denominators for converters were divided in half, since infection was assumed to have occurred halfway during travel. For travellers with a negative TST post-travel and an unknown TST pre-travel, we assumed they were also negative pre-travel. Confidence intervals for both the attack- and incidence rates have been calculated.

## Results

### Study population

**Figure 1:**
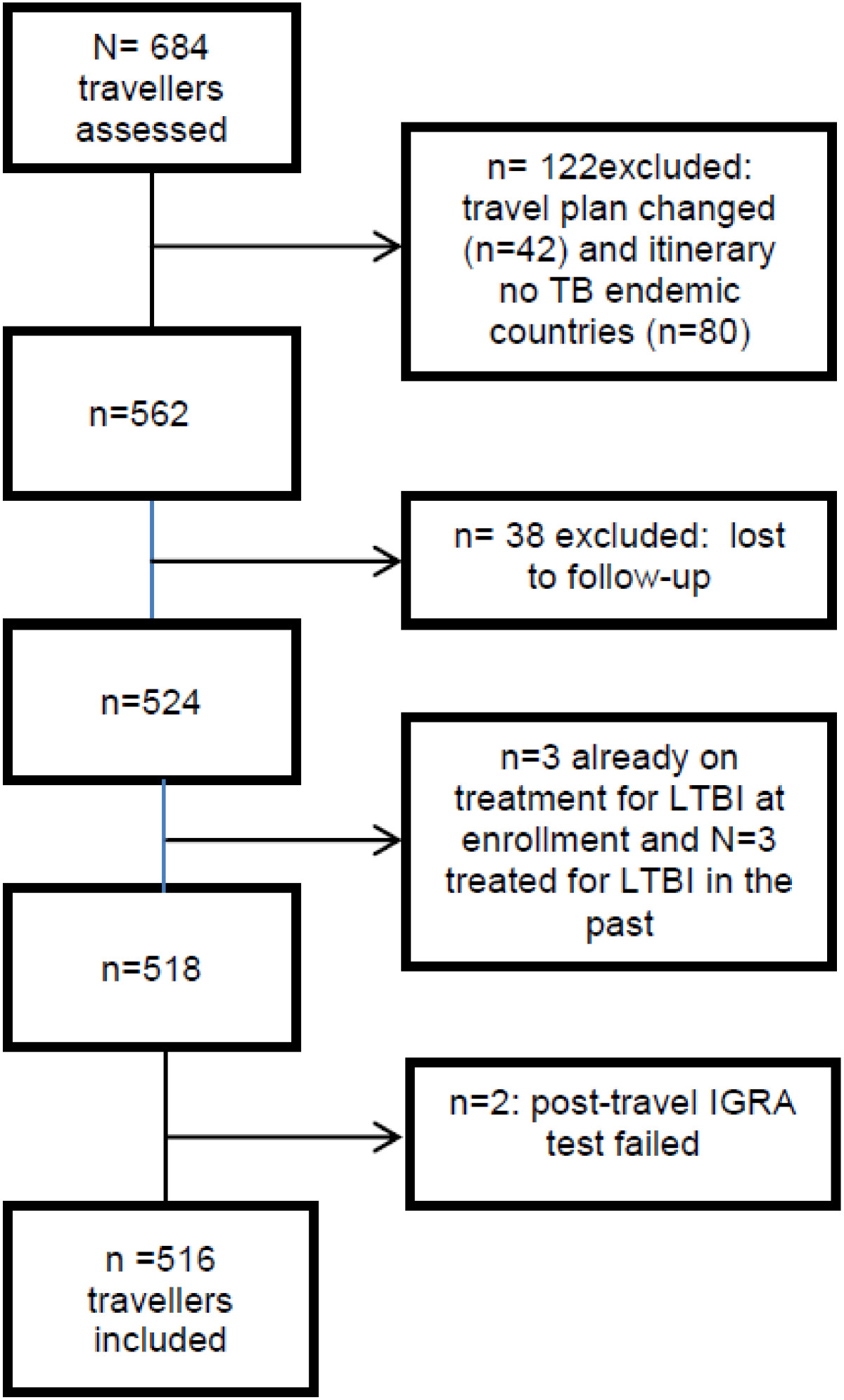
**Flow chart of study population.**

Between December 2008 and September 2011, 684 persons intending to travel to (sub) tropical countries for 13–52 weeks provided informed consent. Of these, 168 were excluded upon completion of the study (Figure 1): 42 travellers had their travel arrangements changed, 80 had an itinerary that did not include TB-endemic countries for ≥13 weeks, 2 were excluded because blood samples failed, 6 were excluded because they received treatment for TB at the time of study (n = 3) or had a positive TST in the past for which treatment had been given (n = 3), and 38 were lost to follow-up. Resulting in a total of 516 travellers included in this study.

### Characteristics

Characteristics are summarised in Table 1. The median age was 25 years and 35% were female. Mean travel duration was 22 weeks. Travel purpose was tourism for 62% of participants, work for 36%, and 2% were visiting friends or relatives (VFR). Of the 187 persons travelling for (volunteer) work, 20 reported work in a medical setting.

**Table 1:** **Characteristics of 516 travelers to TB-endemic countries who attended a Dutch travel clinic for pre-travel health advice; December 2008-September 2011**

		Total	%
**Age**	Median, years (interquartile range)	25.0	(23–30)
**Sex**	Female	336	65
	Male	180	35
**BCG (before travel)**	No	473	92
	Yes	43	8
**Travel history**	Yes	413	80
	No	103	20
**Reason for travel**	Tourism	321	62
	Work: non medical setting	95	18
	Work: medical setting	20	4
	Work: unknown setting	72	14
	VFR	8	2
**Travel duration**	Mean, weeks (SD)	21,9	(8.55)
**Travel destination primary**	South East Asia	190	37
	South America	116	23
	Sub-Saharan Africa	111	22
	Asia, South	50	10
	Caribbean/Central America	29	6
	Asia, other	19	3
	North Africa/Middle East	1	0.2
**Interval between return and blood donation (days)**			
	Mean (SD)	29,8	(16.0)
**Interval between return and TST (days) (n = 261)**			
	Mean (SD)	49.8	(36.8)

TB: tuberculosis; SD: standard deviation.

South East Asia was the primary destination for 37%, South America for 23%, and Sub-Saharan Africa for 22%. Mean interval between return from travel, blood donation and TST was 30 and 50 days respectively.

### Results IGRA

Five of all 516 participants had a positive post-travel IGRA. Of those, three pre-travel samples were already positive, yielding two conversions (Table 2) and resulting in an attack rate of 0.4% (2/513; 95% CI: 0.5 -13.9) and an incidence rate of 0.85 per 1000 person-months (95% CI: 0.1-3.1). Of the tested characteristics, no risk factors predictive for IGRA conversions were found. One traveller, number 7 (Table 2), was initially negative but was tested again later due to a positive TST and appeared then to have seroconverted.

**Table 2:** Positive post-travel IGRA* and TST** results in a cohort of 516 travellers to TB-endemic countries, December 2008-September 2011

Traveller			Travel				IGRA (IU/ml)				TST (mm)				Follow-up at TB department
	Age range (y)	BCG	Travel history	Reason	Destination	Duration (days)	Pre-travel	Post-travel	Interval return and IGRA (days)	Conversion	Pre-travel	Post-travel	Interval return and TST (days)	Conversion	
**1**	31-35	no	Asia: 3w Latin America: 4w	non medical work	SE Asia	365	−0,05	1.15	29	yes	nt	8	39	no	Volunteer work, non-medical. TST performed in 2000: 0 mm. Started preventive treatment 43 days post travel.
**2**	26-30	no	Africa: 1w	tourism	SE Asia	172	−0,03	0,56	25	yes	nt	2	37	no	IGRA repeated 85 days post travel: 0.21 IU/ml. No preventive treatment was started.
**3**	26-30	no	no	non medical work	SE Asia	150	nt	−0,01	27	no	2	13	27	yes	IGRA repeated 55 days post travel: 0.002 IU/ml. According to Dutch guidelines: no preventive treatment was started since IGRA was negative.
**4**	41-45	no	Asia: 3 mo Africa: 5 w	non medical work	SE Asia	356	nt	0,02	28	no	2	15	112	yes	No follow-up.
**5**	18-20	no	no	tourism	SE Asia/ East Asia	93	nt	0,04	38	no	0	11	90	yes	No follow-up IGRA. X-ray 94 days post travel showed no abnormalities; no preventive treatment.
**6**	36-40	no	Asia: 2 mo Latin America: 9 mo	tourism	South Asia	180	nt	−0,05	42	no	0	10	63	yes	No follow-up. No preventive treatment.
**7**	21-25	no	Africa: 6 w	medical setting	Sub-Sahara Africa	118	nt	0,11	18	no	0	10	79	yes	Medical student in a hospital in East-Africa for 3 months. IGRA repeated 79 days post travel: 7.47 IU/ml; Preventive therapy was started.
**8**	26-30	no	Asia: 6 w Latin America: 5 mo	tourism	Central America	147	nt	−0,01	20	no	nt	18	20	unknown	No IGRA follow-up. X thorax showed no abnormalities. No preventive treatment. (TST performed in 2007 was 6 mm).
**9**	41-45	no	Asia: 6 mo Africa: 1 mo	non medical work	sub-Sahara Africa	242	nt	−0,01	51	no	nt	11	51	unknown	No follow-up. According to Dutch guidelines: no preventive treatment was started since IGRA was negative.
**10**	21- 25	no	Africa: 10 mo	tourism	Central America	214	nt	0.00	21	no	nt	10	61	unknown	Advised to have IGRA repeated but no compliance.

IGRA: interferon gamma release assay; TST: tuberculin skin test; TB: tuberculosis; y: years; w: weeks; mo: months; nt: not tested; *: of all 516 samples tested; **: of all 261 TST results.

### Results TST

Of the 516 inclusions, 43 had been BCG-vaccinated. Of the 473 unvaccinated travellers eligible for post-travel TST, 3 had a TST pre-travel result of ≥ 10 mm and 7 travellers received a BCG-vaccine pre-travel. After excluding these 10 participants, 64% (295/463) complied with a post-travel TST, however 34 did not return to let their TST result be read.

Of the remaining 261 post-travel TST, 8 were considered positive. Of these, pre-travel test results were unknown for 3 (non-compliance), resulting in 5 demonstrated and 3 potential conversions (Table 2).

Of the 253 negative post-travel TST 197 had a known negative TST pre-travel. Assuming that the remaining 56 were also negative pre-travel, we found an attack rate (AR) of 1.9% (5/261; 95% CI: 0.6 - 4.4) for demonstrated TST conversions and an incidence rate (IR) of 4.26 per 1000 person-months (95% CI: 1.38- 9.94).

When including potential conversions, we found an AR of 3.1% (8/261) and an IR of 6.76 per 1000 person-months (95% CI: 2.9- 13.3).

None of the tested characteristics were predictive for TST conversion.

### Comparison of IGRA and TST

Of the 2 travellers with an IGRA conversion, one had a TST of 8 mm post-travel (unknown pre-travel TST) and the other was negative (2 mm), tested 39 and 37 days respectively after return (Table 2). The first has been treated for TB infection. The latter had the IGRA test repeated 85 days after return, which was then negative, and was therefore considered not to have TB infection.

All five TST converted individuals and the 3 potential conversions (positive TST post-travel but an unknown pre-travel TST) had a negative post-travel IGRA.

Traveller number 7, who had worked in a hospital in East-Africa, had a negative IGRA 18 days after return. At the TB department 79 days after return the TST was 10 mm. The IGRA at that time was also positive (7.47 IU/ml), and therefore treatment for TB infection was started. This was considered the only confirmed seroconversion; of the remaining 7, none were treated for TB infection.

## Discussion

Based on the IGRA test, this prospective study found a low incidence of recent infection with *M tuberculosis* among long-term immunocompetent travellers to TB-endemic countries; an attack rate (AR) of 0.4% and an incidence rate (IR) of 0.85 per 1000 person-months.

Based on TST conversions, we found an AR of 1.9% and an IR of 4.3 per 1000 person-months comparable to an AR of 1.8% and an IR of 3.5 per 1000 person-months in a previous Dutch study, which had an enrolment period from 1994 till 1996 [1].

Results of IGRA and TST were not concordant as has been found in other studies [8–10]. The most likely reason for a positive TST with a negative IGRA, is a false-positive TST due to atypical mycobacteria. Another explanation for this discrepancy in our study could be that occasionally IGRA was tested earlier than TST. Indeed, in one case in our study (traveller 7, Table 2), a traveller with a negative pre-travel TST who worked in a medical setting in East-Africa for 3 months, had a negative IGRA, tested 18 days post travel. Because of the positive TST 11 weeks post travel, IGRA was repeated at the TB control department and turned out to be highly positive (7.47 IU/ml). This traveller was probably infected in the latter part of his/her stay in East-Africa. This particular case shows that it is important not to perform the IGRA earlier than the TST, which is done at least 8 weeks after return from a TB-endemic country. That this time interval is important is also demonstrated in traveller 2, who showed a positive IGRA tested 3 weeks post travel and a 5 mm TST 5 weeks post travel. The IGRA was repeated 55 days after return and appeared to be negative.

As already mentioned in the introduction, there are several disadvantages of TST compared to IGRA of which the poor compliance is an important one [5]. In addition, compliance for TST was poor in this study at only 56%. A single IGRA visit is expected to increase the compliance for screening and a traveller’s own GP can ask for an IGRA test, which, in contrast to the TST, means that referral to the TB specialist is not necessary. The estimated high specificity of IGRA for the diagnosis of LTBI in immunocompetent subjects justifies that negative results are considered true negatives. However, a positive IGRA requires a physician’s careful risk assessment before deciding to start preventive therapy, which is illustrated by traveller 2 mentioned previously.

The use of IGRA is already known to decrease the number of people that are recommended preventive treatment for TB infection: in the Netherlands adding the IGRA test to the screening protocol (two-step approach of starting with TST, followed by an IGRA if induration of the TST is ≥ 5 mm) has led to a decrease of the number of people who are recommended preventive treatment [11].

Evaluation of the TB department’s follow-up of travellers with a negative IGRA, performed within the incubation period, and a positive TST showed that in most cases no IGRA retesting had taken place, either because the TB physician found the traveller at low risk for TB infection or due to non-compliance. However, none of the participants was registered in the national TB register by the end of 2013. The only traveller in our study who was considered to have a true conversion and who was treated for TB was one of the 20 travellers who reported to have been working in a medical setting. Therefore one might argue whether testing for TB should be limited to only those travellers with potential exposure by working in a medical setting in TB endemic countries.

## Conclusions

We conclude that the risk of acquiring TB infection in immunocompetent long-term travellers is low. A post-travel IGRA alone could be a useful screening tool for TB infection among long-term travellers to high-endemic TB countries, arguably even limited to only those travellers with potential exposure by working in medical settings, enhancing compliance compared to TST. However, the IGRA test should be performed at least 8 weeks after return. A person with a positive IGRA should be referred to a TB physician for further evaluation.

## Authors’ original submitted files for images

Below are the links to the authors’ original submitted files for images. Authors’ original file for figure 1
