# Supplementary material for: Screening travellers to high-endemic countries for infection with Mycobacterium tuberculosis using interferon gamma release assay; a prospective study
Source: BMC Infect Dis. 2014 Sep 23;14:515. doi: 10.1186/1471-2334-14-515 (PMC4179847; doi:10.1186/1471-2334-14-515)
Supplement: Supplementary file 1 — Authors’ original file for figure 1 [file 12879_2014_3831_MOESM1_ESM.pdf]

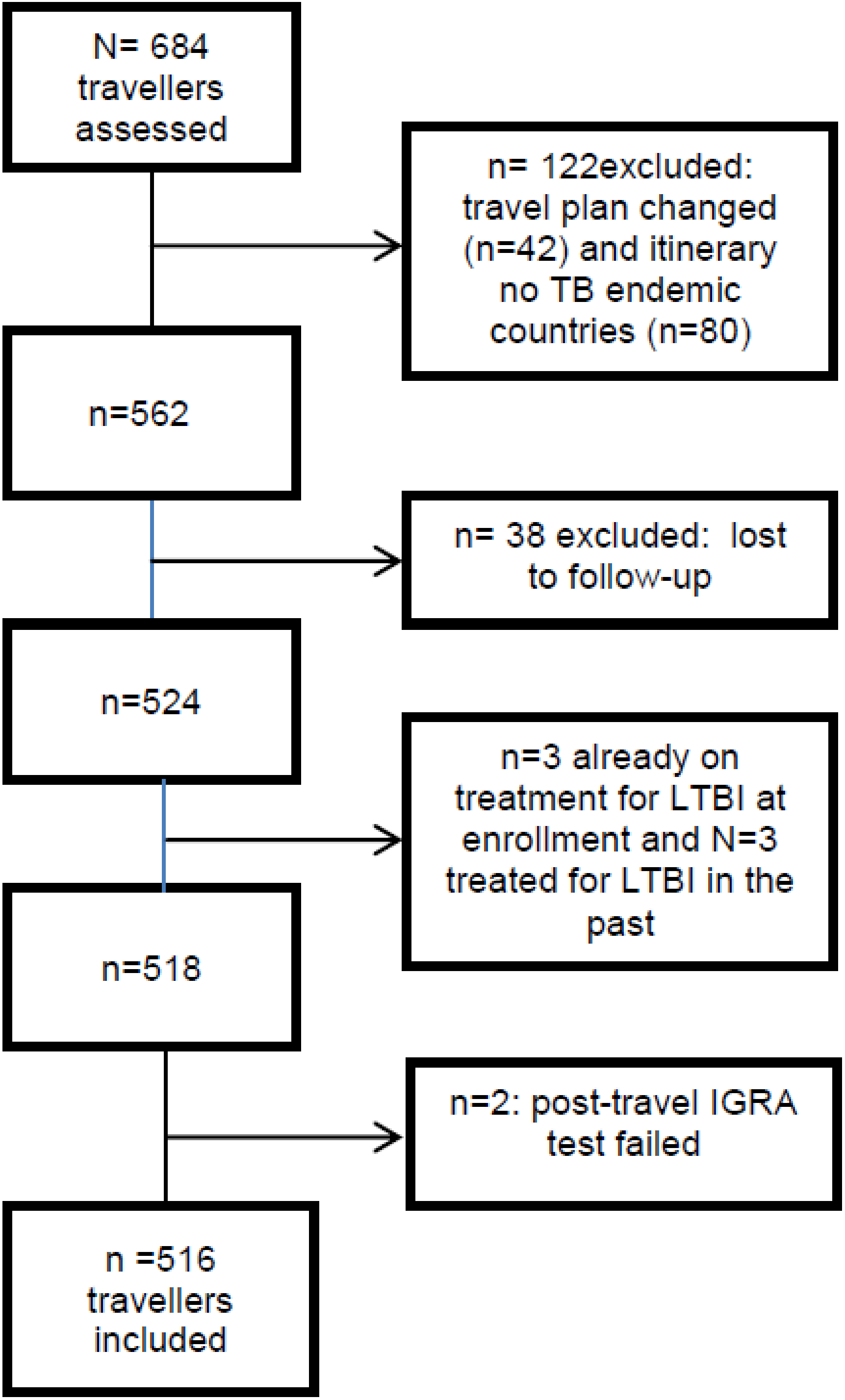
N= 684 travellers assessed
n= 122excluded: travel plan changed (n=42) and itinerary no TB endemic countries (n=80)
n=562
n= 38 excluded: lost to follow-up
n=524
n=3 already on treatment for LTBI at enrollment and N=3 treated for LTBI in the past
n=518
n=2: post-travel IGRA test failed
n =516 travellers included
